# A comparative genome analysis of gene expression reveals different regulatory mechanisms between mouse and human embryo pre-implantation development

**DOI:** 10.1186/1477-7827-8-41

**Published:** 2010-05-11

**Authors:** Kan He, Hongbo Zhao, Qishan Wang, Yuchun Pan

**Affiliations:** 1 School of Agriculture and Biology, Shanghai Jiao Tong University, Shanghai, China; 2 Shanghai Key Lab for Veterinary Biotechnology, Shanghai, 200240, China

## Abstract

**Background:**

Pre-implantation development is a crucial step in successful implantation and pregnancy in mammals. It has been studied in depth, but mostly in laboratory animal models. Less is known about the regulatory mechanism involved in the pre-implantation development in humans and about the comparative aspects.

**Methods:**

Here, we employed the microarray datasets from the public database library of GEO and applied comparative analysis of genome wide temporal gene expression data based on statistical analysis and functional annotation for both mouse and human, demonstrating the discordance between the regulatory mechanisms of both mouse and human pre-implantation development.

**Results:**

There were differences between mouse and human pre-implantation development both in the global gene expression pattern and in the expression changes of individual genes at each stage, including different major transient waves of transcription profiles and some stage-specific genes and significantly related pathways. There also appeared to be different functional changes from one stage to another between mouse and human.

**Conclusions:**

The analysis presented here lead to interesting and complementary conclusions that the regulatory mechanism of human pre-implantation development is not completely the same as the mouse. Not as the fact that 1-cell to 2-cell stage is important for mouse pre-implantation development, the 4-cell stage and 8-cell stage are both essential for human. Unlike in mouse, of which most of pathways found were related to energy, RNA and protein metabolism, the identified pathways in human were mostly disease-related and associated with human pre-implantation embryonic development. All of these suggest that a further comparative analysis should be required for applying the result of mouse expression data to human research or therapy, particularly in pre-implantation developments. Our study provides several potential targets of genes and pathways for studying the regulatory mechanism of human pre-implantation development using mouse model.

## Background

Pre-implantation development is a mammalian-specific occurrence, which encompasses the period from fertilization to implantation and involves a number of important events [1]. Understanding pre-implantation development is important, both for basic reproductive biology and for practical applications including regenerative medicine and livestock production. However, due to the scarcity of the materials, both in size (about 0.1 mm diameter) and in quantity (only a few to tens of oocytes from each ovulation) are limited in related research, which has hampered the molecular analysis of human pre-implantation embryos. Thus using the mouse model system has formed the current perfect paradigm about gene expression during pre-implantation development. Recently, more and more global gene expression profiles during mouse pre-implantation development have been examined, two principal transient waves of de novo transcription have been identified [2,3]. Additionally, several important transcripts have been reported to have the core roles at each developmental stage. For example, *H1foo *and *Msy2 *have been reported as the oocyte-specific transcripts, which are not re-expressed later in development, destruction of these maternal mRNAs restricts the length of time that these genes can function [4,5]; Recent studies have shown that *JNK *and *p38 *are involved in cavity formation during pre-implantation development [6,7]. As the unclear confidence of the previously identified genes, their roles in the regulation of mouse pre-implantation development must be further discussed and the consistence with human must also be considered.

In our study, we employed time course expression datasets of early mouse and human embryo both from the same series. Through our comparative analysis of genome wide temporal gene expression data based on significance analysis and functional annotation, we found that there were many differences in the expression patterns of pre-implantation development between mouse and human, for both the regulatory waves and the identified genes.

## Methods

### Data collection

We searched GEO [8] for the gene expression profiling studies related to pre-implantation development. Finally, we chose the data set GSE18290 contributed by Xie D, containing 52 samples of early bovine embryo, human embryo, and mouse embryo [9]. As we were interested in the mouse and human, we deleted 16 bovine samples. There were 36 samples left, 18 for human (GSM456643 to GSM456660) and 18 for mouse (GSM456661 to GSM456678). Human and mouse embryos were harvested at successive stage from oocyte to blastocyst. They were both generated at one-cell stage, two-cell stage, four-cell stage, eight-cell stage, morula, and blastocyst, the number of replication is 3. Total RNAs were extracted, amplified and hybridized onto Affymetrix microarrays. The platforms were Affymetrix Mouse Expression 430A Array (MOE430A, total 22690 probe sets) and Affymetrix Human Genome U133 Plus 2.0 Array (HG-U133_Plus_2, total 54675 probe sets).

### Microarray data analysis

The datasets we chose (CEL files) were downloaded from GSE18290. For mouse and human respectively, 18 samples were divided into 6 groups according to different periods (the time condition). Probe intensities were then normalized, and expression signals of all genes (probe sets) were calculated using the Robust Multichip Averaging (RMA) algorithm in affy package [10,11]. Statistical analysis was performed by one-way ANOVA with a Benjamini and Hochberg False Discovery Rate (BH-FDR = 0.05) for multiple testing correction followed by Tukey's post-hoc tests [12]. Differentially expressed genes between two neighbor stages were identified by 2.0 fold-changes. Clustering on groups and genes was performed based on the identified genes' expression using the method of Hierarchical clustering. All of these processes were performed using software packages developed in version 2.4.0 of Bioconductor and R version 2.10.0 [10,13].

### GO annotations and pathway analysis

Further classifications of GO annotations and pathway analysis were performed by using the Database for Annotation, Visualization and Integrated Discovery (DAVID) [14,15], revealing over-represented function of identified genes associated with developmental stages or time specification.

## Results and Discussion

### Global outlook by time-course analysis

**Figure 1:**
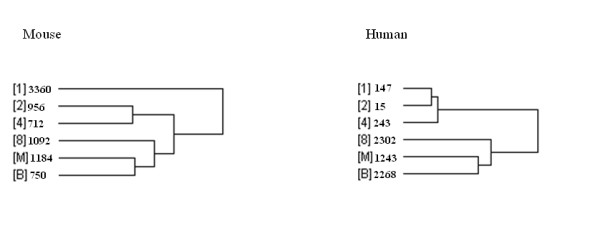
**The comparison of Hierarchical clustering analysis on samples' groups between mouse and human**. Hierarchical clustering analysis showed the similarity in transcription profiles among the samples tested for mouse and human [1,2,4,8], [M] and [B] denote the samples' groups of one-cell stage, two-cell stage, four-cell stage, eight-cell stage, morula and blastocyst, respectively.

Through our analysis by ANOVA-FDR test with False Discovery Rate (FDR) ≤ 5%, 12930 probe sets were shown statistically significant changes during mouse pre-implantation development. By contrast, the number for human was 21746. To identify the gene expression patterns of these two species, we employed hierarchical clustering on the samples' groups by using the above identified genes and then had a comparison between mouse and human (Figure 1). Obviously, there was difference between mouse and human transcription profiles. For mouse, we identified two major transitions in the gene expression patterns: one-cell stage to two-cell stage and 4-cell stage to 8-cell stage. These transitions separated mouse pre-implantation embryos into three phases: one-cell stage (Mouse Phase 1); two-cell stage and 4-cell stage (Mouse Phase 2); and 8-cell stage, morula and blastocyst (Mouse Phase 3). The current microarray data were mostly consistent with the previously reported expression patterns of genes [2]. By contrast, for human, there were also two major transitions, but they were: 4-cell stage to 8-cell stage and 8-cell stage to morula. Similarly, they separated human pre-implantation embryos into three phases: one cell stage, two-cell stage and 4-cell stage (Human Phase 1); 8-cell stage (Human Phase 2); morula and blastocyst (Human Phase 3).

**Figure 2:**
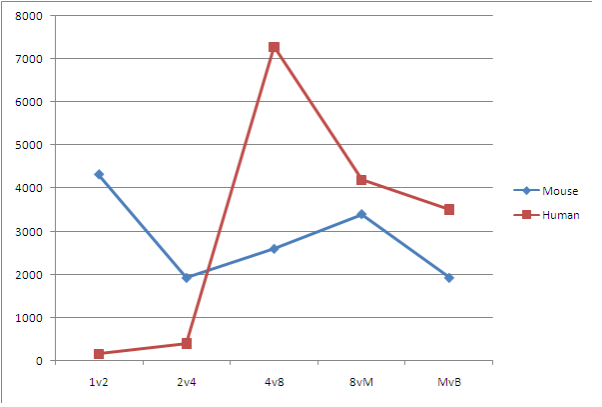
**The trends of number of significantly regulated genes during pre-implantation development between mouse and human**. Mouse and human trends of significantly regulated genes number during pre-implantation development are depicted by blue and red, respectively. X axis represents 5 different stage comparisons of pre-implantation development, including from 1-cell to 2-cell (1v2); from 2-cell to 4-cell (2v4); from 4-cell to 8-cell (4v8); from 8-cell to Morula (8 vM); from Morula to Blastocyst (MvB). Y axis represents the number of significantly regulated genes. The result is based on the data in Table 1.

To obtain a perspective on global gene expression changes, we performed a pair-wise comparison of gene expression data for all pre-implantation developmental stages. The threshold of Fold Change was 2.0. The results for mouse and human are detailed in Table 1 and Figure 2. The lists of mouse and human significantly regulated genes in each stage comparison can be seen in Additional file 1 and 2, respectively. For mouse, there were two major transient waves of gene expression changes. The first wave was from one-cell stage to two-cell stage, the number of identified differently expressed probe sets was totally 4316, including 1867 down-regulated and 2449 up-regulated. The second one was from 8-cell stage to morula, a total of 3400 probe sets were identified, 819 for down-regulated and 2581 for up-regulated. In comparison, only one major wave appeared in human expression profiles, but the appearing periods focused on the later stages. It was from 4-cell stage to 8-cell stage, total number is 7278 including 2180 down-regulated and 5098 up-regulated. These found waves were mostly consistent with the result of above hierarchical clustering, also revealing different gene activations among pre-implantation developmental stages between mouse and human.

**Table 1:** The comparisons of gene expression between embryo stages for mouse and human

FC ≥ 2.0		1 v 2	2 v 4	4 v 8	8 v M	M v B
**Mouse**	**down**	1867	1242	1233	819	1794
	**up**	2449	691	1368	2581	140
**Human**	**down**	10	375	2180	2423	1979
	**up**	152	30	5098	1768	1532

1, 2, 4, 8, M and B denote one-cell stage, two-cell stage, four-cell stage, eight-cell stage, morula, and blastocyst, respectively. 1 v 2 represents the comparison of gene expression between one-cell stage and two-cell stage, down is for the down-regulated genes and up is for the up-regulated genes. FC represents the threshold of Fold Changes = 2.0. The number represents the identified genes' count of each comparison for mouse and human.

Our present finding suggests that in 1- and 2- cell stages of humans, there are not many genes that get regulated. But subsequently, numerous of the differentially expressed genes have been identified in both 4-cell stage and 8-cell stage, thus these two stages may play essential roles in human pre-implantation development. The most early and widely cited report has also shown that some of the major qualitative changes which occur between the four- and eight-cell stages are dependent on transcription and cleavage is not sensitive to transcriptional inhibition until after the four-cell stage [16]. Moreover, according to more biological processes participating in Human Phase 3 (morula and blastocyst), it suggests that more preparations should be needed for implantation of human. On the contrary, in the case of mouse though most of the genes are regulated (up or down) between 1- and 2-cell stages, there are fewer but essential genes up/down regulated during later stages of pre-implantation development. The transition from the two-cell to four-cell stage is particularly important in pre-implantation mouse embryonic development, as it involves transcriptional reprogramming and cellular differentiation [17]. It is well known that the transcriptome of the mammalian embryo is comprised of maternally deposited transcripts after fertilization, but maternal transcripts are degraded and replaced by zygotic transcripts after several cell divisions, the transition is called zygote genome activation (ZGA) [2,3]. Our findings suggest that the timing of ZGA and maternal transcripts degradation is different between mice and human, it occurs between 1-cell and 2-cell stage in mouse, but between 4-cell and 8-cell stage in human development. They are supported by previous reports [2,16,18].

### Analysis of individual genes

**Figure 3:**
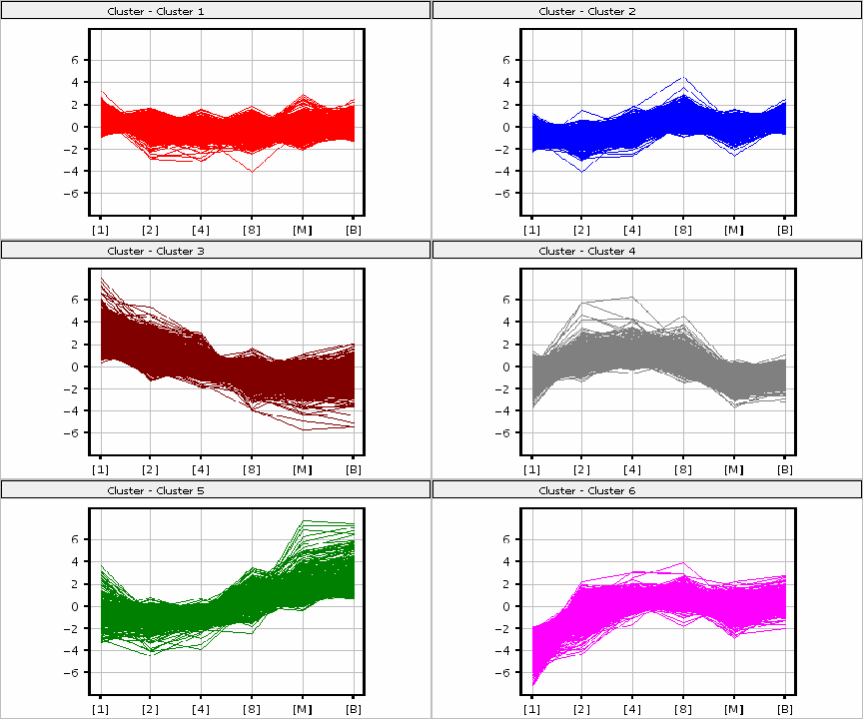
**K-means clustering on significant genes for mouse**. General trends of expression changes were analyzed by k-means clustering method for mouse. 6 clusters were classified and represented by different colors. X axis represents 6 different stages of pre-implantation development; y axis represents log intensities of individual genes.

**Figure 4:**
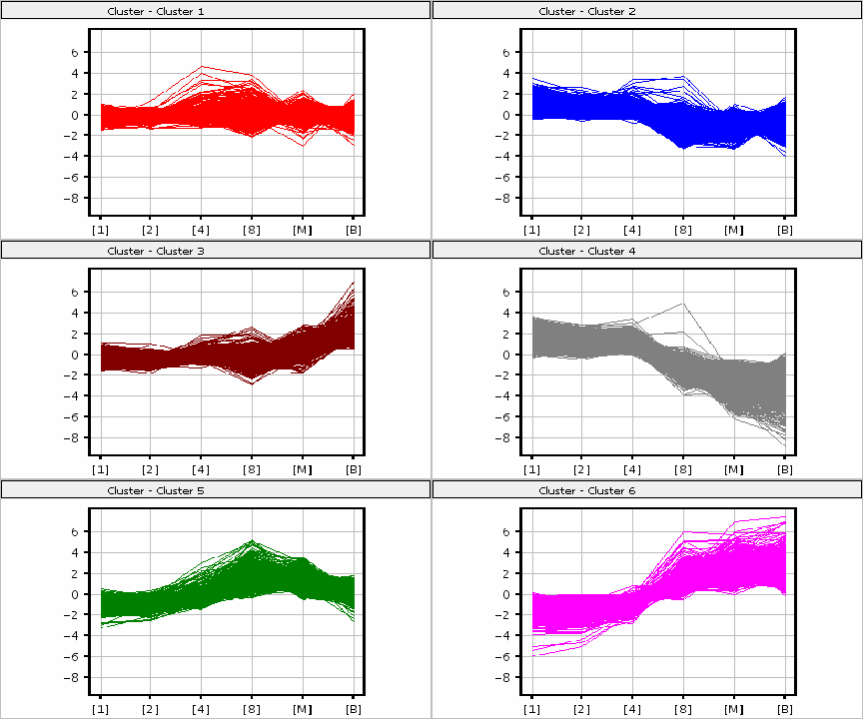
**K-means clustering on significant genes for human**. General trends of expression changes were analyzed by k-means clustering method for human. 6 clusters were classified and represented by different colors. X axis represents 6 different stages of pre-implantation development; y axis represents log intensities of individual genes.

In order to provide information about the expression changes of individual genes over time, we further respectively analyzed 12930 and 21746 statistically significant genes by a k-means clustering method for mouse and human pre-implantation development [19]. Although the dynamics of actual gene expression changes of individual genes was very complex, the k-means clustering provided a good overview of expression trends and formed a wave-like expression pattern. As a result, six clusters were identified respectively for mouse and human (Figure 3 and 4). The significant genes from each cluster were shown in Additional File 3 and 4. Several regulated genes specific to each cluster were different between mouse and human.

For mouse, the identified 6 clusters can be assigned to four main groups. The first group appears to represent genes that showed a steady increase and decrease throughout pre-implantation stages, including cluster 1 (containing 5749 probe sets) and cluster 2 (2197 probe sets). In this group, it contains *Esrra *and *Esrrb *respectively in cluster 2 and cluster 1, which have been reported as associated with obesity and type 2 diabetes [20,21]. The second group appears to represent genes that showed a dramatic decrease throughout the stages, abundant in one cell stage but degraded during pre-implantation development, which is cluster 3 (1883 probe sets). The third group is exactly opposite to the second one, it appears to represent genes that showed a dramatic increase throughout the developmental stages, including cluster 5 (690 probe sets). The last group includes cluster 4 (1721 probe sets) and cluster 6 (690 probe sets), the genes here were firstly activated from one cell stage to 8-cell stage, peaked at 8-cell stage but degraded at later stages. In fact, the last group contains *Mapk1 *and *Mapk3*, respectively in cluster 4 and cluster 6, which are both well-known genes having central roles in diverse cellular functions [22-25].

For human, these 6 clusters can also be divided into 4 groups. The first group appears to represent genes that showed a steady increase and decrease through the stages, except from 4-cell stage to 8-cell stage. The cluster 1 (containing 9169 probe sets) showed a dramatic increase from 4-cell stage to 8-cell stage, whereas the cluster 2 (5751 probe sets) showed a dramatic decrease during the period. In this group, there are our interesting genes like *ESR1 *and *ESR2*. As in the mouse, The second group appears to represent genes that showed a dramatic decrease throughout the stages, abundant in one cell stage but degraded during pre-implantation development, which is cluster 4 (1907 probe sets). The third group appears to represent genes that showed a dramatic increase throughout the developmental stages, including cluster 3 (2214 probe sets) and cluster 6 (894 probe sets). The last group only includes cluster 5 (1811 probe sets), also like the mouse, appearing to represent genes that were firstly activated from one cell stage to 8-cell stage, peaked at 8-cell stage but degraded at later stages. For central genes, we also take the mitogen activated protein kinase (MAPK) members for example. *Mapk1 *includes in cluster 2, 3 and 5; there are *Mapk7 *in cluster 2, *Mapk8 *in cluster 4, *Mapk9 *in cluster 3, *Mapk12 *in cluster 2, *Mapk13 *in cluster 1 and *Mapk14 *in cluster5. Almost all of MAPK members involve in the regulation of human pre-implantation development.

During mouse pre-implantation development, the exclusive expression of the *Xist *gene from the paternally inherited allele is thought to play a role in the inactivation of the paternally inherited X chromosome in the extra-embryonic cell lineages of the developing female embryo. The previous study using human cleavage-stage embryos derived by in vitro fertilization revealed that a pattern of *XIST *expression different from that in the mouse [26]. In our study, the profiles of *XIST *expression in both mouse and human pre-implantation development were known. As a result, the expression of *Xist *was significantly regulated at mouse 2-cell stage and blastocyst, but almost significantly regulated through human pre-implantation development (from 2-cell to blastocyst). On the other hand, in the k-means clustering of significant genes, the mouse *Xist *gene was in Cluster 5 but the human *XIST *gene was in Cluster 3. The details were shown in Additional file 1 and 2, colored in red. Thus, all of these demonstrate that not as in the mouse, the pattern of human *XIST *expression is not consistent with a role for early expression in the choice of paternal X inactivation but participates in regulating the whole human early embryo development.

### Significantly related GO terms and pathways

We then sought to determine the functions associated with the identified significant genes between each adjacent stage, analysis of over-represented annotations and pathways were performed by using DAVID [14,15]. If p value was less than 0.01, it was considered as significant GO annotation or pathway. The significant GO terms of over-represented genes were shown in Table 2 and 3 respectively for mouse or human. The significant pathways of over-represented genes were shown in Table 4 and 5 respectively for mouse or human.

**Table 2:** The over-represented classification of GO annotations for mouse identified genes

	GO	p value
**1 v 2** **(16)**	GO:0044237~cellular metabolic process	1.29E-36
	GO:0044238~primary metabolic process	1.10E-35
	GO:0043170~macromolecule metabolic process	2.51E-31
	GO:0006412~translation	1.47E-24
	GO:0006396~RNA processing	7.26E-17
	GO:0009058~biosynthetic process	1.11E-15
	GO:0016043~cellular component organization and biogenesis	4.25E-10
	GO:0033036~macromolecule localization	9.42E-10
	GO:0045184~establishment of protein localization	2.45E-08
	GO:0051236~establishment of RNA localization	7.03E-08
	GO:0051649~establishment of cellular localization	1.81E-07
	GO:0051641~cellular localization	1.94E-07
	GO:0009719~response to endogenous stimulus	3.33E-07
	GO:0007049~cell cycle	4.72E-06
	GO:0007059~chromosome segregation	2.76E-05
	GO:0022402~cell cycle process	2.74E-04
	GO:0051301~cell division	2.08E-03
**2 v 4** **(13)**	GO:0006412~translation	3.45E-25
	GO:0044237~cellular metabolic process	2.41E-23
	GO:0044238~primary metabolic process	3.32E-23
	GO:0043170~macromolecule metabolic process	2.39E-20
	GO:0009058~biosynthetic process	3.38E-15
	GO:0006396~RNA processing	2.37E-09
	GO:0045184~establishment of protein localization	4.15E-06
	GO:0033036~macromolecule localization	5.72E-06
	GO:0009056~catabolic process	2.71E-05
	GO:0016043~cellular component organization and biogenesis	7.18E-05
	GO:0051236~establishment of RNA localization	1.14E-03
	GO:0051641~cellular localization	1.21E-03
	GO:0051649~establishment of cellular localization	1.55E-03
**4 v 8** **(18)**	GO:0044238~primary metabolic process	1.36E-12
	GO:0044237~cellular metabolic process	6.67E-11
	GO:0043170~macromolecule metabolic process	1.09E-10
	GO:0016043~cellular component organization and biogenesis	1.02E-08
	GO:0051301~cell division	3.26E-08
	GO:0051641~cellular localization	9.22E-08
	GO:0051649~establishment of cellular localization	2.26E-07
	GO:0009058~biosynthetic process	2.41E-07
	GO:0006412~translation	6.46E-06
	GO:0006396~RNA processing	6.77E-06
	GO:0007049~cell cycle	6.78E-06
	GO:0033036~macromolecule localization	8.58E-06
	GO:0045184~establishment of protein localization	2.35E-05
	GO:0022402~cell cycle process	2.49E-05
	GO:0009719~response to endogenous stimulus	1.82E-03
	GO:0007059~chromosome segregation	3.13E-03
	GO:0009056~catabolic process	3.62E-03
	GO:0065009~regulation of a molecular function	7.05E-03
**8 v M(17)**	GO:0044238~primary metabolic process	1.92E-32
	GO:0044237~cellular metabolic process	1.80E-31
	GO:0006396~RNA processing	4.57E-25
	GO:0006412~translation	2.48E-24
	GO:0043170~macromolecule metabolic process	2.54E-21
	GO:0009058~biosynthetic process	5.20E-18
	GO:0016043~cellular component organization and biogenesis	1.61E-11
	GO:0033036~macromolecule localization	2.59E-09
	GO:0045184~establishment of protein localization	8.07E-09
	GO:0051236~establishment of RNA localization	1.36E-08
	GO:0051649~establishment of cellular localization	5.39E-08
	GO:0051641~cellular localization	6.48E-08
	GO:0051301~cell division	1.12E-06
	GO:0009056~catabolic process	1.25E-04
	GO:0007049~cell cycle	2.38E-04
	GO:0009719~response to endogenous stimulus	4.92E-04
	GO:0022402~cell cycle process	2.11E-03
**M v B (15)**	GO:0006412~translation	3.01E-21
	GO:0009058~biosynthetic process	9.93E-21
	GO:0044237~cellular metabolic process	4.03E-15
	GO:0006396~RNA processing	4.06E-13
	GO:0044238~primary metabolic process	8.43E-13
	GO:0033036~macromolecule localization	3.53E-12
	GO:0016043~cellular component organization and biogenesis	9.61E-10
	GO:0045184~establishment of protein localization	1.35E-09
	GO:0043170~macromolecule metabolic process	1.03E-08
	GO:0051641~cellular localization	5.79E-08
	GO:0051649~establishment of cellular localization	2.05E-07
	GO:0006091~generation of precursor metabolites and energy	1.36E-06
	GO:0051236~establishment of RNA localization	2.51E-05
	GO:0009056~catabolic process	9.86E-05
	GO:0051301~cell division	1.37E-03

Classification of GO of Biological Process was done with DAVID using the genes identified over-represented between each adjacent stage for mouse. P value less than 0.01 was identified as significant. The number in the bracket represents the count of significant GO terms.

**Table 3:** The over-represented classification of GO annotations for human identified genes

	GO	p value
**1 v 2** **(4)**	GO:0051301~cell division	1.92E-03
	GO:0044237~cellular metabolic process	3.40E-03
	GO:0006396~RNA processing	6.67E-03
	GO:0022402~cell cycle process	8.89E-03
**2 v 4** **(4)**	GO:0044238~primary metabolic process	1.67E-03
	GO:0044237~cellular metabolic process	2.92E-03
	GO:0016265~death	5.13E-03
	GO:0006396~RNA processing	8.17E-03
**4 v 8** **(23)**	GO:0043170~macromolecule metabolic process	2.72E-45
	GO:0044238~primary metabolic process	1.47E-38
	GO:0044237~cellular metabolic process	2.95E-34
	GO:0006396~RNA processing	7.68E-27
	GO:0016043~cellular component organization and biogenesis	1.00E-25
	GO:0019222~regulation of metabolic process	2.41E-18
	GO:0050794~regulation of cellular process	3.30E-18
	GO:0010468~regulation of gene expression	1.21E-17
	GO:0006350~transcription	2.63E-17
	GO:0007049~cell cycle	7.90E-16
	GO:0051301~cell division	4.09E-15
	GO:0033036~macromolecule localization	6.39E-14
	GO:0050789~regulation of biological process	1.18E-12
	GO:0045184~establishment of protein localization	1.36E-11
	GO:0051236~establishment of RNA localization	5.45E-11
	GO:0022402~cell cycle process	5.56E-10
	GO:0051649~establishment of cellular localization	1.10E-07
	GO:0007059~chromosome segregation	2.18E-07
	GO:0051641~cellular localization	5.55E-07
	GO:0006412~translation	4.44E-05
	GO:0051656~establishment of organelle localization	5.55E-04
	GO:0006376~mRNA splice site selection	1.24E-03
	GO:0016265~death	5.38E-03
**8 v M** **(21)**	GO:0044238~primary metabolic process	6.11E-25
	GO:0044237~cellular metabolic process	2.20E-24
	GO:0043170~macromolecule metabolic process	4.54E-19
	GO:0016043~cellular component organization and biogenesis	3.52E-10
	GO:0006412~translation	5.04E-08
	GO:0006396~RNA processing	1.20E-06
	GO:0051236~establishment of RNA localization	2.12E-06
	GO:0007049~cell cycle	4.24E-06
	GO:0009058~biosynthetic process	7.48E-06
	GO:0009719~response to endogenous stimulus	7.65E-06
	GO:0022402~cell cycle process	1.01E-05
	GO:0033036~macromolecule localization	1.21E-05
	GO:0045184~establishment of protein localization	6.87E-05
	GO:0009056~catabolic process	7.37E-04
	GO:0007059~chromosome segregation	7.54E-04
	GO:0051301~cell division	1.01E-03
	GO:0051649~establishment of cellular localization	3.81E-03
	GO:0019222~regulation of metabolic process	3.92E-03
	GO:0019748~secondary metabolic process	3.97E-03
	GO:0051641~cellular localization	4.23E-03
	GO:0050794~regulation of cellular process	5.35E-03
**M v B** **(21)**	GO:0044238~primary metabolic process	6.38E-14
	GO:0044237~cellular metabolic process	1.65E-13
	GO:0043170~macromolecule metabolic process	5.25E-09
	GO:0016043~cellular component organization and biogenesis	6.25E-08
	GO:0033036~macromolecule localization	2.08E-07
	GO:0050794~regulation of cellular process	7.93E-07
	GO:0045184~establishment of protein localization	4.81E-06
	GO:0050789~regulation of biological process	6.99E-06
	GO:0051641~cellular localization	3.08E-05
	GO:0051649~establishment of cellular localization	6.51E-05
	GO:0010468~regulation of gene expression	1.68E-04
	GO:0019222~regulation of metabolic process	2.30E-04
	GO:0016265~death	4.69E-04
	GO:0006396~RNA processing	5.29E-04
	GO:0007049~cell cycle	6.10E-04
	GO:0006350~transcription	1.46E-03
	GO:0022402~cell cycle process	1.71E-03
	GO:0051301~cell division	5.49E-03
	GO:0009058~biosynthetic process	5.64E-03
	GO:0048468~cell development	6.89E-03
	GO:0009056~catabolic process	9.26E-03

Classification of GO of Biological Process was done with DAVID using the genes identified over-represented between each adjacent stage for human. P value less than 0.01 was identified as significant. The number in the bracket represents the count of significant GO terms.

**Table 4:** The over-represented classification of pathways for mouse identified genes

	pathways	p value
**1 v 2** **(10)**	mmu03010:Ribosome	1.25E-40
	mmu04120:Ubiquitin mediated proteolysis	4.56E-08
	mmu00240:Pyrimidine metabolism	6.90E-06
	mmu00230:Purine metabolism	8.55E-05
	mmu04110:Cell cycle	1.25E-04
	mmu03050:Proteasome	2.07E-04
	mmu03020:RNA polymerase	3.18E-03
	mmu04115:p53 signaling pathway	3.71E-03
	mmu00190:Oxidative phosphorylation	3.87E-03
	mmu03030:DNA polymerase	7.14E-03
**2 v 4** **(7)**	mmu03010:Ribosome	2.02E-39
	mmu03050:Proteasome	3.67E-09
	mmu04110:Cell cycle	6.60E-04
	mmu00040:Pentose and glucuronate interconversions	1.93E-03
	mmu00190:Oxidative phosphorylation	3.33E-03
	mmu00230:Purine metabolism	4.03E-03
	mmu00500:Starch and sucrose metabolism	9.65E-03
**4 v 8** **(5)**	mmu03010:Ribosome	2.50E-08
	mmu03050:Proteasome	4.84E-07
	mmu00190:Oxidative phosphorylation	2.41E-05
	mmu04120:Ubiquitin mediated proteolysis	2.63E-03
	mmu00020:Citrate cycle (TCA cycle)	3.94E-03
**8 v M** **(10)**	mmu03010:Ribosome	1.01E-25
	mmu03050:Proteasome	2.73E-13
	mmu00240:Pyrimidine metabolism	2.26E-10
	mmu03020:RNA polymerase	1.23E-08
	mmu04110:Cell cycle	1.97E-07
	mmu00230:Purine metabolism	1.36E-05
	mmu00500:Starch and sucrose metabolism	4.42E-04
	mmu04120:Ubiquitin mediated proteolysis	6.82E-04
	mmu00040:Pentose and glucuronate interconversions	8.00E-04
	mmu00190:Oxidative phosphorylation	4.44E-03
**M v B** **(9)**	mmu03010:Ribosome	4.82E-19
	mmu00190:Oxidative phosphorylation	2.53E-16
	mmu03050:Proteasome	1.17E-09
	mmu00280:Valine, leucine and isoleucine degradation	6.28E-06
	mmu00020:Citrate cycle (TCA cycle)	1.21E-05
	mmu04110:Cell cycle	5.17E-04
	mmu00100:Biosynthesis of steroids	3.50E-03
	mmu00071:Fatty acid metabolism	3.50E-03
	mmu00281:Geraniol degradation	4.47E-03

Classification of related pathways was done with DAVID using the genes identified over-represented between each adjacent stage for mouse. P value less than 0.01 was identified as significant. The number in the bracket represents the count of significant pathways.

**Table 5:** The over-represented classification of pathways for human identified genes

	pathways	p value
**1 v 2** **(0)**	none	none
**2 v 4** **(3)**	hsa04540:Gap junction	2.29E-03
	hsa00100:Biosynthesis of steroids	2.84E-03
	hsa05060:Prion disease	4.75E-03
**4 v 8** **(8)**	hsa04110:Cell cycle	3.37E-09
	hsa04120:Ubiquitin mediated proteolysis	1.44E-07
	hsa04520:Adherens junction	5.58E-06
	hsa04115:p53 signaling pathway	2.87E-04
	hsa03022:Basal transcription factors	8.31E-04
	hsa00230:Purine metabolism	4.87E-03
	hsa05215:Prostate cancer	4.99E-03
	hsa00970:Aminoacyl-tRNA biosynthesis	8.41E-03
**8 v M** **(6)**	hsa04120:Ubiquitin mediated proteolysis	9.93E-05
	hsa04110:Cell cycle	1.58E-04
	hsa05131: Eenteropathogenic E. coli (EPEC) infection	3.82E-03
	hsa05130: Enterohemorrhagic E. coli (EHEC) infection	3.82E-03
	hsa04115:p53 signaling pathway	3.93E-03
	hsa05221:Acute myeloid leukemia	7.63E-03
**M v B** **(11)**	hsa00100:Biosynthesis of steroids	2.26E-06
	hsa05131: Eenteropathogenic E. coli (EPEC) infection	5.82E-05
	hsa05130: Enterohemorrhagic E. coli (EHEC) infection	5.82E-05
	hsa04120:Ubiquitin mediated proteolysis	1.78E-04
	hsa00251:Glutamate metabolism	3.03E-04
	hsa04520:Adherens junction	7.05E-04
	hsa00071:Fatty acid metabolism	2.75E-03
	hsa05110:Cholera - Infection	2.93E-03
	hsa00280:Valine, leucine and isoleucine degradation	6.04E-03
	hsa05211:Renal cell carcinoma	6.26E-03
	hsa00900:Terpenoid biosynthesis	7.14E-03

Classification of related pathways was done with DAVID using the genes identified over-represented between each adjacent stage for human. P value less than 0.01 was identified as significant. The number in the bracket represents the count of significant pathways.

With comparison to mouse, due to the less different identified genes from one-cell stage to two-cell stage and from two-cell stage to 4-cell stage for human, there were less GO terms during these periods. The GO terms for human focused on such as "cell division", "cellular metabolic process", "primary metabolic process" and "RNA processing". These were all obtained for mouse, except for the GO term "death". Just like to be delayed, most of previous GO terms for mouse were appearing from 4-cell to 8-cell stage for human, such as "macromolecule metabolic process", "macromolecule localization", "establishment of protein localization" and "establishment of RNA localization". These findings support the idea that mid-preimplantation gene activation (MGA) drives the overt morphological changes in the subsequent stages, compaction and bifurcation into two-cell lineages. With the preparation of basic cellular machinery, dramatic biological processes were happening during Human Phase 3 (morula and blastocyst), which is related to the event of implantation.

Briefly, through our pathway analysis, most of the identified pathways were involved in after human 4-cell stage, but the number of identified pathways shared in mouse pre-implantation development. Moreover, many of the features and signaling pathways that are required during human pre-implantation development are also active during tumourigenesis [27]. According to our analysis based on human data, p53 signaling pathway, the pathways of Eenteropathogenic E. coli (EPEC) infection and Enterohemorrhagic E. coli (EHEC) infection were identified separately in 4-cell and 8-cell stages. These pathways were also thought to be related to the formation of cancer and the regulation of human reproduction [28-31]. But these above pathways were not identified from microarray analysis in mouse 4-cell and 8-cell stages. On the contrary, the pathway of oxidative phosphorylation was mostly identified through mouse pre-implantation development, which hardly appeared in human data. The oxidative phosphorylation pathway was reported as one of the obligatory energy metabolism pathways in most species throughout pre-implantation development [32]. And the pathway of Citrate cycle (TCA cycle) was identified after mouse 8-cell stage, which was not identified in human data. It is well know that the mitochondrial TCA cycle is the major source of reducing equivalents in the cytosol so that any change in mitochondrial function in the embryo will be reflected in changes in the intracellular redox state. In the mouse, the metabolic substrates used by the oocyte and early embryo each have a different impact on the intracellular redox state [33]. Surprisingly, the common identified pathway from 4-cell to 8-cell stage between human and mouse was only the Ubiquitin mediated proteolysis pathway, which was reported as an important role in eukaryotic cellular processes [34]. Thus it is advisable to choose the Ubiquitin mediated proteolysis pathway as the candidate pathway for further study on reproductive biology and regenerative medicine using mouse model. Interestingly, some of the known metabolic pathways e.g. biosynthesis of steroids pathway and fatty acid metabolism pathway were shown significant in both human and mouse blastocysts. All of these suggest that unlike in mouse, of which most of pathways found were related to energy, RNA and protein metabolism, the identified pathways in human were mostly disease-related and associated with human pre-implantation embryonic development. By contrast, there are some common metabolic pathways participate in regulating the mammal early embryonic development.

Furthermore, in view of the fact that many researchers working in mice interpret their results in relation to human reproduction, mice and human reproduction differ distinctly in many aspects; e.g. the reproduction in mice is characterized by a very short oestrus cycle and thus, displays a distinctly different endocrine dynamic pattern if compared with humans or domestic animals [35-38]. On the other hand, due to the difference in the inner environment of embryo and the external environment of uterus, there are species differences in the implantation process between mice and human [39-41]. The same applies for the regulatory mechanisms of early embryonic development that can be not simply transferable to the human species.

In sum, pre-implantation development involves a number of biologically significant events, such as compaction and blastocyst formation, which represent morphologically dynamic changes, especially for mouse and human [27]. Although the models of mouse have been well examined, molecular mechanisms regulating the early embryo development of human have been scarcely reported and the credibility of using the mouse model to explain the regulatory mechanism of human pre-implantation development remains unclear. Briefly, in this study, we have shown differences between mouse and human pre-implantation developments both in the global gene expression pattern and the expression changes of individual genes at each stage, including different major transient waves of transcription profiles and some stage-specific genes and pathways. Undeniably, Studies in mice have provided insights into the molecular basis of human pre-implantation development because of their shared features. In both species, early embryo development leads to a complex regulatory mechanism. However, the nature of human embryonic signals that influence uterine functions is more difficult than rodents', especially at the later stages of pre-implantation development (morula and blastocyst). Thus, it limits the availability of adequate amounts of tissues for mouse analysis. Moreover, the quality and quantity of samples chosen in microarray experiments also play essential roles in effecting the accuracy of mouse model, especially the conditions for in vitro maturation and fertilization are crucial for the proper early embryo development and may result in developmental aberrations.

## Conclusions

By comparison between mouse and human expression profiles, we have noted that the regulatory mechanism of human pre-implantation development is different from the mouse, and even more complex. Through our analysis, we have found differences between mouse and human transcription profiles both in the global expression pattern of genes and in expression of individual genes within the gene clusters identified and in the significantly related pathways. Not as the fact that 1-cell to 2-cell stage is important for mouse pre-implantation development, the 4-cell stage and 8-cell stage are both essential for human. Unlike in mouse, of which most of pathways found were related to energy, RNA and protein metabolism, the identified pathways in human were mostly disease-related and associated with human pre-implantation embryonic development. Different expression patterns and significantly related biological processes during each stage between mouse and human suggest that a further comparative analysis should be required for applying the result of mouse expression data to human research or therapy, particularly in pre-implantation developments. Our study also provides several potential targets of genes and pathways for studying the regulatory mechanism of human pre-implantation development using mouse model, such as the gene of *Xist *and the Ubiquitin mediated proteolysis pathway.

## Competing interests

The authors declare that they have no competing interests.

## Authors' contributions

KH designed the study, collected the datasets from databases and analyzed the data, then wrote the manuscript. QSW is acknowledged for excellent technical assistance with text data mining and statistical algorithm. HBZ designed the bioinformatics study and reviewed the manuscript. YCP designed the study and reviewed the manuscript. All authors read and approved the final manuscript.

## Supplementary Material

Additional file 1 **The list of mouse significantly regulated genes in each stage comparison**. The data provided represent the list of mouse significantly regulated genes in each stage comparison, including from 1-cell to 2-cell (1v2); from 2-cell to 4-cell (2v4); from 4-cell to 8-cell (4v8); from 8-cell to Morula (8 vM); from Morula to Blastocyst (MvB), and containing the information of probe set IDs, Fold change, regulation type (down or up), gene symbols and gene titles. Click here for file

Additional file 2 **The list of human significantly regulated genes in each stage comparison**. The data provided represent the list of human significantly regulated genes in each stage comparison, including from 1-cell to 2-cell (1v2); from 2-cell to 4-cell (2v4); from 4-cell to 8-cell (4v8); from 8-cell to Morula (8 vM); from Morula to Blastocyst (MvB), and containing the information of probe set IDs, Fold change, regulation type (down or up), gene symbols and gene titles. Click here for file

Additional file 3 **significant genes of K means clustering for mouse**. The data provided represent the list of significant genes in 6 clusters for mouse, containing probe set IDs, gene symbols and gene titles. Click here for file

Additional file 4 **significant genes of K means clustering for human**. The data provided represent the list of significant genes in 6 clusters for human, containing probe set IDs, gene symbols and gene titles. Click here for file
